# Optimization of hybrid polymer preparation by ex situ embedding of waste Fe/Mn oxides into chitosan matrix as an effective As(III) and As(V) sorbent

**DOI:** 10.1007/s11356-019-05856-x

**Published:** 2019-07-05

**Authors:** Daniel Ociński

**Affiliations:** Department of Industrial Chemistry, Wroclaw University of Economics, ul. Komandorska 118/120, 53-345 Wrocław, Poland

**Keywords:** Arsenic, Hybrid polymers, Adsorption, Iron oxides, Chitosan

## Abstract

A hybrid polymer for deep removal of arsenic from aqueous solutions was obtained by loading of waste Fe/Mn oxides into a chitosan matrix. The process was optimized by studying the influence of selected individual factors and their reciprocal combinations on the adsorptive and physical properties of the product. The influence of chitosan solution concentration, inorganic load amount, the ratio of Fe/Mn oxides to chitosan, and polymer cross-linking degree on kinetics of As(III) and As(V) adsorption was examined. The optimal values of the parameters were chitosan polymer concentration 1.5% *w*/*w*, inorganic load to chitosan ratio 1.67, and glutaraldehyde to chitosan amine groups molar ratio 3:1. The selected products were evaluated in terms of their morphology (scanning electron microscopy (SEM) with EDS analysis), porosity (N_2_ and CO_2_ adsorption isotherms), surface properties (Fourier-transform infrared spectroscopy (FTIR), isoelectric point determination) and durability in an acidic environment. The proposed process makes it possible to obtain a product combining beneficial adsorptive properties toward arsenic with the physical form and durability essential in fixed-bed adsorption systems.

**Electronic supplementary material:**

The online version of this article (10.1007/s11356-019-05856-x) contains supplementary material, which is available to authorized users.

## Introduction

The utilization of industrial wastes containing iron or aluminum oxides as the sorbents for various micropollutants present in natural waters and wastewaters has received much attention. Such wastes, derived mainly from water treatment (water treatment residuals—WTRs) or bauxite processing (red mud—RM), usually exhibit advantageous adsorptive properties toward heavy metals, metalloids, and dyes (Giles et al. 2011; Jacukowicz-Sobala et al. 2015; Cusack et al. 2019). Special effort has been given to the removal of arsenates and arsenites due to their toxic and carcinogenic properties for humans (Mohammed Abdul et al. 2015). Arsenic is an ubiquitous element in the Earth’s crust and enters natural waters primarily through weathering of rocks. Arsenic occurs in the aqueous environment mainly in the form of arsenates and more toxic arsenites. Long-term ingestion of water with an elevated concentration of arsenic compounds causes a variety of diseases, including chronic health effects and cancers (Huang et al. 2015). This problem affects many parts of the world, including some Asiatic countries, the USA, and the European Union (Shakoor et al. 2017; Ghosh et al., 2019).

Most studies on the use of industrial wastes as arsenic sorbents were focused on by-products from coagulation processes taking place in water treatment plants (Jacukowicz-Sobala et al. 2015). Only a few studies have dealt with the use of residuals from groundwater deironing, which exhibit uniquely advantageous properties (Gibbons and Gagnon 2010; Ociński et al. 2016b). Since this process is based on water aeration aimed at oxidizing Fe^2+^ to Fe^3+^, the obtained sludge consists mainly of iron hydroxides and oxo-hydroxides. Moreover, due to the presence of Mn(II) in the treated groundwater, MnO_2_ is created during the oxidation, resulting in a change of properties of the formed sludge, which acquires oxidizing properties. Since arsenites exist in natural waters mainly in the form of undissociated molecules (pK_a1_ = 9.2, pK_a2_ = 12.1, pK_a3_ = 13.4), which are less effectively adsorbed than arsenates (pK_a1_ = 2.2, pK_a2_ = 7.1, pK_a3_ = 11.5) (Fig. S1), the presence of MnO_2_ contributes—due to the ability to oxidize As(III) to As(V)—to enhancement of the overall adsorption effectiveness (Zhu et al. 2015a; Ociński et al. 2016b).

Nonetheless, the physical form of these residuals (small particle size) causes difficulties in the implementation of the process (separation of the spent sorbent, high hydraulic resistance in fixed-bed systems). Embedding of the waste sludge in the porous matrix of organic polymers leads to obtaining hybrid materials composed of a macromolecular skeleton and inorganic deposit. Due to the combination of the properties of both constituents, it is possible to obtain a sorbent exhibiting high mechanical strength and advantageous hydraulic properties ensured by a porous polymeric matrix, simultaneously showing high adsorptive properties characteristic for inorganic oxides (Cumbal et al. 2003; Sarkar et al. 2012; Samiey et al. 2014; Smith et al. 2015). However, the majority of hybrid polymers was synthesized by in situ methods based on sol–gel processes starting with the introduction of precursors (metal salts) into the preformed polymer matrix containing functional groups enabling even distribution of the metal precursor and followed by hydrolysis occurring within the polymeric matrix. This process was applied for the formation of various types of hybrid polymers (Cumbal and Sengupta 2005; Jacukowicz-Sobala et al. 2013; Acelas et al. 2015; Feng and Sun 2015; Taleb et al. 2015; Kociołek-Balawejder et al. 2017). However, these methods are inappropriate for deposition of the preformed particles. The incorporation of wasted oxides into the polymeric matrix has to be based on ex situ methods, which include deposition of preformed microparticles of an inorganic load within the polymer matrix. This can be achieved by dispersion of metal oxide particles in the solution of the polymer, followed by its precipitation (occurring as a consequence of pH change or cross-linking) resulting in impregnation of the used matrix. Natural polymers such as alginate, chitosan, or modified cellulose, due to their availability, low-cost, and easy processing, are often used as the supporting materials (Wang and Chen 2014; Basu et al. 2015; Lee et al. 2017; López-García et al. 2017; Basu et al. 2018; Kumar et al. 2018; Ociński et al. 2019). Special attention has been focused on the use of chitosan in the formation of hybrid sorbents, owing to its high reactivity and beneficial physical properties. Chitosan is a transformed polysaccharide obtained by *N*-deacetylation of chitin and contains both hydroxyl and amino groups with significant adsorption potential for anionic and cationic species. Moreover, the presence of functional groups ensures excellent surface hydrophilic properties of chitosan and enables cross-linking of its linear chains, which improves the stability of the polymer in the acidic environment.

While there are many studies concerning the use of chitosan for hybrid sorbents’ preparation (Miller and Zimmerman 2010; Seyed Dorraji et al. 2014; Wang et al. 2014; Qi et al. 2015; Gerard et al. 2016; He et al. 2016; Martínez-Cabanas et al. 2016; Su et al. 2016; Dhoble et al. 2017; López-García et al. 2017), there is a lack of a detailed and thorough survey on the influence of selected process parameters on adsorptive and physical properties of the obtained material. Aside from the native properties of the used inorganic deposit, the stability and adsorptive properties of the formed hybrid polymer result from the composition and the sorbent’s structure, its swelling capacity in aqueous solutions, and cross-linking degree of the polymeric chains. The main goal of this study was to examine the influence of the individual forming parameters, as well as their reciprocal combinations, on the stability and sorptive properties of the hybrid polymer obtained by ex situ embedding of residuals from water deironing into a chitosan polymer matrix.

## Experimental

### Materials

The waste Fe/Mn oxides (Mn-WTRs) from water deironing were collected from “Na Grobli” Water Treatment Plant, Wrocław, Poland. The detailed characterization of this by-product was presented elsewhere (Ociński et al. 2016b). The raw sludge was rinsed several times with distilled water, pre-treated with 0.1 M HCl, dried, ground, and sieved (0.25-mm sieve). All reagents used in this study were of analytical grade. Medium molecular weight chitosan, sodium (meta)arsenite (NaAsO_2_), and disodium hydrogen arsenate (Na_2_HAsO_4_·7H_2_O) were obtained from Sigma-Aldrich; glutaraldehyde solution (25% *w*/*w*) used as the cross-linking agent was acquired from Chempur, Poland.

### Formation of the hybrid polymer

The hybrid polymer was formed by encapsulation of the pre-treated waste Fe/Mn oxides into chitosan followed by cross-linking of the product. By using various polymer solution concentrations and inorganic load amounts, various ratios of chitosan to Mn-WTRs and different amounts of cross-linking agent, 15 variants of hybrid polymer, with different physical and adsorptive properties, were obtained (Table 1). The overall formation procedure is as follows. The proper amounts of chitosan were mixed vigorously with 0.1 M HCl solution for 24 h at 50 °C until complete dissolution of the polymer. Then, after cooling to room temperature, the suspension of well-dispersed Fe/Mn oxides in water was added to the polymer solution and stirred until complete homogenization was achieved (1 h). In the next step, the spherical beads of hybrid polymer were prepared via drop-wise addition of the suspension into 0.5 M NaOH solution using a peristaltic pump (Gilson MiniPuls3) equipped with a micropipette tip (0.5 mm in diameter) placed at the end of the dispensing tube. The product was separated and washed several times with distilled water to a neutral pH. A part of the obtained beads was then contacted with glutaraldehyde solution for 18 h, washed with distilled water, and stored for further use. The successive studies were conducted using both wet (for all variants of hybrid polymer) and air-dried sorbent (for products selected on the basis of preliminary adsorption experiments).

**Table 1:** Characteristics of the formed hybrid polymers

Product	Chitosan (CH)		Mn-WTRs		Mn-WTRs/CH ratio	Glutaraldehyde (GA)		Bead’s characterization	
	m, g	c, % (*w*/*w*)	m, g	c, % (*w*/*w*)		GA:CH	*V*_5% GA_, mL	mass, mg	Fe content (%)
CWTR-1						–	–	1.013	16.90
CWTR-2	1.76	1.5	2.94	2.5	1.67	1:1	7.56	1.013	16.90
CWTR-3						3:1	22.8	1.053	16.26
CWTR-4						–	–	0.853	20.65
CWTR-5	1.18	1.0	2.94	2.5	2.50	1:1	5.04	0.853	20.65
CWTR-6						3:1	15.12	0.880	20.03
CWTR-7						–	–	1.493	23.27
CWTR-8	1.18	1.0	5.88	5.0	5.00	1:1	4.8	1.507	23.06
CWTR-9						3:1	14.4	1.507	23.06
CWTR-10						–	–	1.800	22.64
CWTR-11	1.76	1.5	5.88	5.0	3.33	1:1	7.44	1.853	21.99
CWTR-12						3:1	22.26	1.920	21.22
CWTR-13						–	–	1.187	16.54
CWTR-14	2.06	1.75	2.94	2.5	1.43	1:1	8.64	1.293	15.17
CWTR-15						3:1	25.92	1.360	14.43

### Characterization of the hybrid polymer

The morphology of the dried beads and their chemical composition were analyzed using a scanning electron microscope (HITACHI S-3400N) equipped with energy-dispersive spectrometry (EDS) microanalyzer (Thermo Scientific Ultra Dry). Specific surface area and the porous characteristics of the sorbent were determined from the adsorption isotherms for liquid nitrogen at 77 K (using Brunauer–Emmett–Teller (BET) and Barrett–Joyner–Halenda (BJH) methods) as well as for CO_2_ at 273 K (for micropores in the range 0.4–2.0 nm) with an Porosimetry Analyzer (ASAP 2020, Micromeritics). The infrared spectra of the bare cross-linked chitosan and Mn-WTRs loaded chitosan were collected on a Fourier transform, Bruker VERTEX 70V vacuum spectrometer equipped with an air-cooled DTGS detector.

The content of iron in the sorbent beads was analyzed in duplicate by contacting 10 wet beads or 0.025 g of dried sorbent with 25 mL of HCl 1:1 for 24 h. The obtained solutions, after dilution, were analyzed in terms of iron content. The mass of the formed beads was determined by air-drying of 25 beads for 72 h and weighing. This procedure was repeated three times, and the average mass of a single bead was determined. The point of zero charge (pH_pzc_) of the hybrid polymer was determined by contacting a 0.025-g sample with 0.1 M KCl solutions (25 mL) at different initial pH (4–9). After 24 h, the final pH of each solution was measured, a graph of the initial pH vs ΔpH was plotted, and the pH_pzc_ value was determined by the point at which the curve crossed pH axis (Newcombe et al. 1993). The analysis was conducted in duplicate. The durability of selected products in acidic conditions was studied by contacting a 0.025-g sample of the dried beads with 25 mL of 0.001, 0.05, 0.01, and 0.1 M HCl solutions for 24 h followed by analysis of dissolved iron.

### Adsorption studies

Adsorption experiments were performed in duplicate, in a batch regime, separately for As(III) and As(V). Preliminary studies were conducted for all 15 variants of the hybrid polymer in its wet form, by contacting 5 wet beads with 50 mL of arsenic solution (10 mg/L) for 17 h. Kinetics were studied by contacting 25 wet beads of the sorbent with 50 mL of arsenic solution (10 mg/L). For the selected product, the adsorption kinetics was performed by contacting 0.025, 0.05, or 0.1 g of the dried beads with 25 mL of arsenic solution (4 mg As/L) for 72 h.

### Analytical methods

The concentration of arsenic in the treated solutions was determined using the molybdenum blue method with the absorbance measurement at 905 nm (Dhar et al. 2004) using a UV–Vis spectrophotometer (Specord 210, Analytik Jena). Because this method is ineffective toward arsenites, prior to the analysis, potassium iodate was used to convert As(III) into As(V). The concentrations of iron in solutions were analyzed using the spectrophotometric thiocyanate method (Specord 210, Analytik Jena).

## Results and discussion

### Determination of key factors influencing sorptive and physical properties of the formed hybrid polymer

Among basic factors influencing both the formation process and the final properties of the obtained sorbent, the following are the most significant: the acid used for chitosan dissolution and the polymer solution concentration, the content of inorganic deposit in the product, the ratio of inorganic deposit to polymeric support, and the cross-linking degree of the polymer. The proper combination of the above factors is necessary to obtain a product with desired adsorptive properties and physical form applicable in fixed-bed adsorption processes. Unlike in most studies of chitosan formation reported in the literature where acetic acid (1–5% *w*/*w*) was used, in this work, the polymer was dissolved in 0.1 M HCl solution. Former studies confirmed only slight weight loss of Fe/Mn oxides during rinsing with such a solution (Ociński et al. 2016a). The concentrations of the used chitosan solutions were 1.0, 1.5, and 1.75% (*w*/*w*). The decrease in polymer concentration below 1% resulted in the formation of unstable and labile beads, whereas the concentration exceeding 1.75% caused difficulties in the homogenization of Fe/Mn oxide dispersion due to the high viscosity of the obtained mixture. The content of the Mn-WTRs in the prepared suspension was 2.5 and 5.0% *w*/*w* and was chosen on the basis of the previous studies dealing with the entrapment of waste Fe/Mn oxides into alginate beads (Ociński et al. 2016a). As a result, various hybrid polymers have been obtained which were characterized by different ratios of inorganic deposit to chitosan matrix between 1.43 and 5.0 (Table 1).

**Fig. 1:**
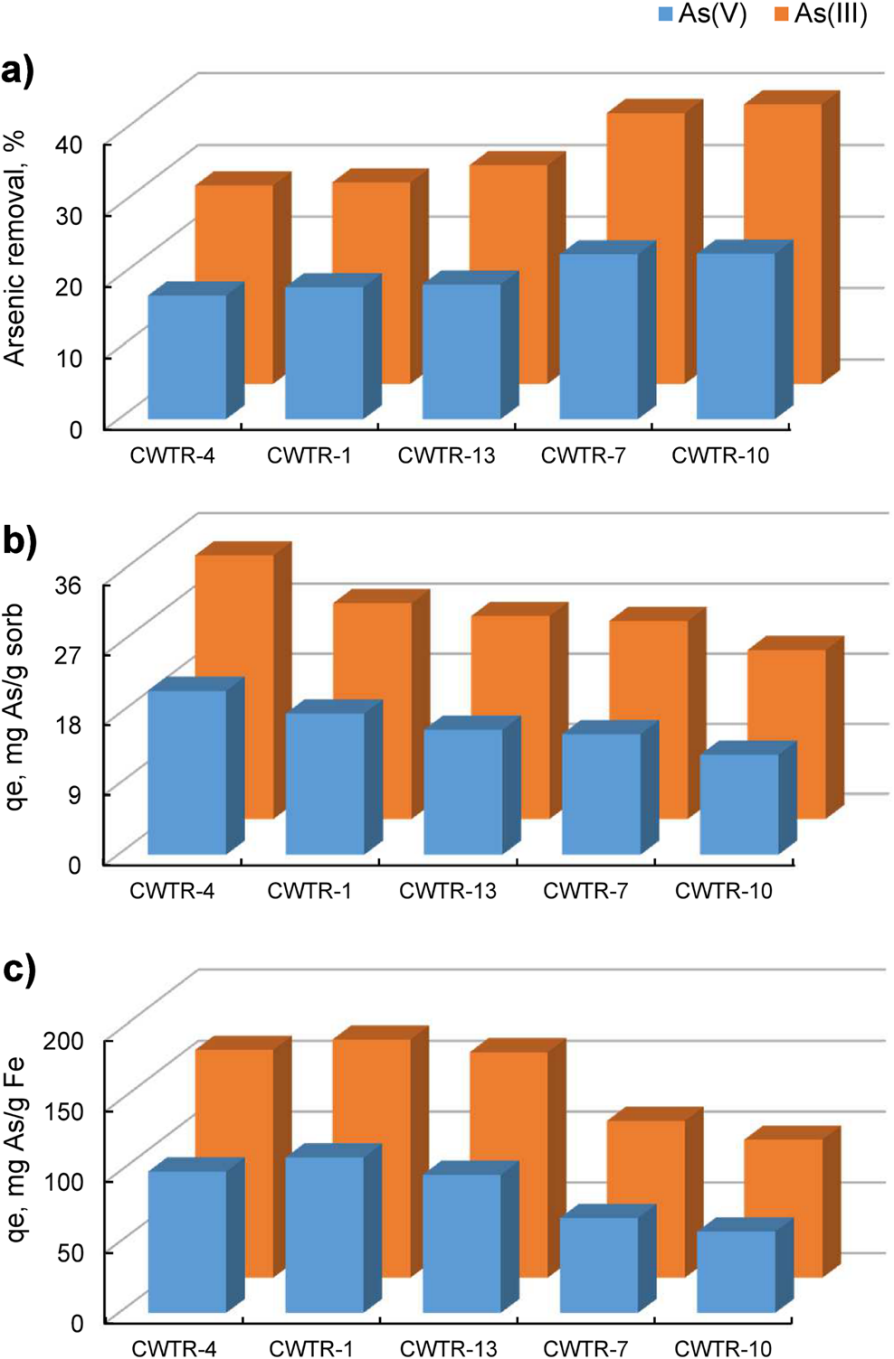
Arsenic adsorption effectiveness for selected hybrid polymers in the wet form: *c*_0_ = 10 mg/L, *t* = 17 h, 5 sorbent beads

The results of preliminary, simplified adsorption studies are presented in Fig. 1. Since adsorption was carried out under neutral pH conditions, its effectiveness was noticeably better for arsenites, due to a different mechanism of their binding on the surface of iron/manganese oxides (Ociński et al. 2016b). Arsenate adsorption proceeds with the formation of inner-sphere complexes on the surface of iron oxides, which is assisted by electrostatic attraction between H_2_AsO_4_^−^/HAsO_4_^2−^ and the sorbent surface. At a pH close to the pH_pzc_ of the sorbent (~ 7.0), its surface becomes negative, which suppresses the adsorption of anions. On the contrary, arsenites occur under such conditions in the form of undissociated molecules (Fig. S1) so that the repulsions are minimized. Moreover, arsenites are first oxidized in the presence of manganese oxides, which is accompanied by Mn^2+^ release:

1 $$ {\mathrm{Mn}\mathrm{O}}_2+{\mathrm{H}}_3{\mathrm{AsO}}_3+{\mathrm{H}}_3{\mathrm{O}}^{+}\to {\mathrm{Mn}}^{2+}+{\mathrm{H}}_2{{\mathrm{AsO}}_4}^{-}+2{\mathrm{H}}_2\mathrm{O} $$

Manganese cations adsorb on the sorbent surface giving it a positive charge, which facilitates the sorption of the formed As(V) oxyanions (Zhang et al. 2007; Kong et al. 2014; Zhu et al. 2015b).

**Fig. 2:**
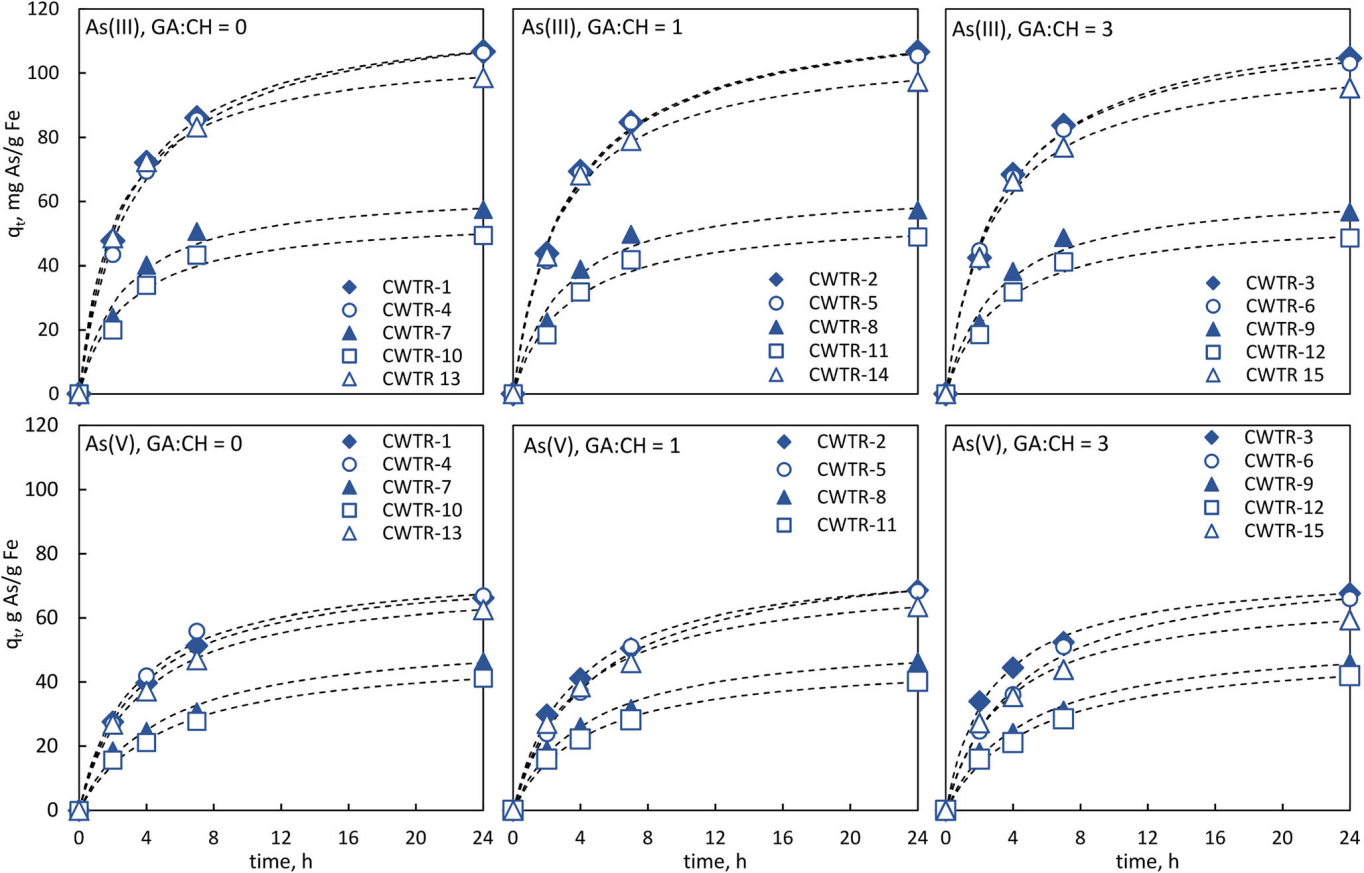
Kinetic curves for As(III) and As(V) adsorption on the formed hybrid polymers: *c*_0_ = 10 mg/L, *t* = 24 h, 25 sorbent beads, the dashed lines are the plots of pseudo-second-order kinetic model

Naturally, the most visible drop in arsenic concentration was observed for products containing the largest amount of inorganic load (CWTR-7, CWTR-10), but the utilization degree of iron oxides (expressed in mg As/g Fe) was significantly higher for polymers with a lower Mn-WTR/chitosan ratio, for both As(III) and As(V). This may be explained by the physical form of the sorbent. Since the most readily available adsorption sites are located on the bead surface, together with the increase of inorganic load content inside the beads, the degree of their exploitation decreases. To investigate this issue more closely, all formed products have been cross-linked using both a stoichiometric (1:1) and an excessive amount (3:1) of glutaraldehyde in relation to amine groups present in chitosan chains. Then, kinetic studies were conducted using all obtained products (CWTR-1 to CWTR-15). The experimental data were analyzed using pseudo-first-order and pseudo-second-order kinetic reaction models (Table S1). The results, presented in Fig. 2 and Table 2, showed almost no effect of cross-linking degree on adsorption kinetics and the overall adsorption efficiency.

**Table 2:** Pseudo-second-order (PSO) adsorption kinetic parameters for arsenic adsorption on the formed hybrid polymers in the wet form

Product	As(III)					As(V)				
	*R* ^2^	*q*_m_. mg/g	*q*_exp_. mg/g	*k*_2_. g/(mg·h) (× 10^−3^)	*h*_0_. mg/(g·h)	*R* ^2^	*q*_m_. mg/g	*q*_exp_. mg/g	*k*_2_. g/(mg·h) (× 10^−3^)	*h*_0_. mg/(g·h)
CWTR-1	0.9997	119.22	106.73	3.00	42.91	0.9998	75.82	66.14	4.25	23.74
CWTR-2	0.9993	121.12	106.67	2.75	37.93	0.9994	78.54	68.54	3.75	21.97
CWTR-3	0.9990	118.81	104.62	2.75	37.31	0.9994	74.96	67.60	5.00	27.81
CWTR-4	0.9990	120.58	106.31	2.75	38.22	0.9978	76.15	66.82	4.25	23.74
CWTR-5	0.9984	120.05	105.34	2.75	36.90	0.9990	81.83	68.24	2.75	17.40
CWTR-6	0.9997	116.10	103.07	3.00	38.80	0.9984	77.80	65.91	3.00	18.35
CWTR-7	0.9961	64.14	57.47	6.25	24.64	0.9955	55.25	46.38	3.75	11.48
CWTR-8	0.9953	64.75	57.30	5.50	22.17	0.9974	54.39	46.35	4.25	12.55
CWTR-9	0.9953	64.18	56.61	5.25	21.35	0.9970	54.59	45.90	4.0	11.56
CWTR-10	0.9957	55.42	49.36	6.75	20.12	0.9972	49.74	41.25	4.0	9.75
CWTR-11	0.9953	55.91	48.95	5.75	17.36	0.9988	47.43	40.20	5.0	10.70
CWTR-12	0.9957	55.46	48.58	5.75	17.36	0.9969	50.73	41.96	4.0	9.87
CWTR-13	0.9996	107.46	98.47	4.50	49.99	0.9997	71.75	62.50	4.0	19.99
CWTR-14	0.9992	108.45	97.35	3.50	40.23	0.9991	72.86	63.47	3.75	19.91
CWTR-15	0.9994	106.31	95.41	3.50	39.21	0.9987	67.55	59.34	4.5	19.66

Only for arsenic(III) can a very slight correlation be observed—the increase in cross-linking degree caused the gradual decrease in kinetic parameters (*k*_2_, *h*_0_) and adsorption capacity (*q*_m_, *q*_exp_). In the case of As(V), there was no such explicit reliance. On the other hand, it is well known from the literature that cross-linking of chitosan diminishes its sorptive properties toward anionic species due to depletion of amine groups reacted with glutaraldehyde. Although the imine formed can still undergo protonation, the chains of glutaraldehyde attached to the nitrogen atom impeded the diffusion of removing species into the active sites (Ruiz et al. 2001; Jóźwiak et al. 2017). Nonetheless, it applies to the pure chitosan used as the sorbent, which binds the removed species by means of their functional groups. In the case of the studied hybrid polymers, their adsorptive properties are provided mainly by the inorganic load, whereas chitosan acts as the supporting material ensuring desirable mechanical properties of the sorbent’s beads. Owing to that, the cross-linking of the chitosan chains leads to an improvement of mechanical properties of the sorbent without any substantial loss in its adsorptive capacity. The observed slight negative effect of cross-linking on arsenite adsorption may arise from impeded transport of removed species into the beads. As arsenates are adsorbed mainly on the beads’ surface, this effect is not noticeable for As(V). As shown in Table 2, the values of the kinetic constant *k*_2_ for As(V) adsorption were almost equal for all hybrid polymers, whereas in the case of arsenite sorption, these values were around twice as high for sorbents containing a double amount of inorganic load (CWTR-7, CWTR-10). Furthermore, the decrease of the amount of inorganic deposit dispersed within the polymer matrix, causing the reduction of bead diameter (Fig. S2), resulted in greater exploitation of Fe/Mn oxides, especially in the process of arsenite adsorption. The maximum adsorption capacity, normalized by the iron content (expressed as mg As/g Fe), increased almost by double for As(III) and only about 50% for As(V). Thus, it may confirm the lower ability of arsenates to diffuse into the sorbent beads. To sum up, taking into consideration the adsorptive properties of the formed beads, especially *q*_m_ and the initial sorption rate *h*_0_—which is chiefly important in column processes—it seems that products CWTR-1 to CWTR-6 are the most promising for further studies. However, as the studied hybrid polymers exhibited differential shape stability and consistency during adsorption experiments, and are intended to be used in the dried form in fixed-bed adsorption systems, their behavior during air-drying has also been studied. Hydrogels in the wet form, mainly due to the low mechanical properties, are unsuitable in fixed-bed processes. As can be seen in Fig. S2, the cross-linking of the polymer matrix substantially influenced its ability to form spherical and uniform beads. All non-cross-linked products underwent deformation while drying, the greater the higher the ratio of inorganic load to chitosan was. Additionally, drying of the products containing only 1% of chitosan, even cross-linked, caused their deformation and fracturing (CWTR-4 to CWTR-9). So, having regard to adsorptive and physical properties of the formed hybrid polymers, the products CWTR-1 to CWTR-3 were selected for further studies using dried beads.

### Characterization of selected hybrid polymers

**Fig. 3:**
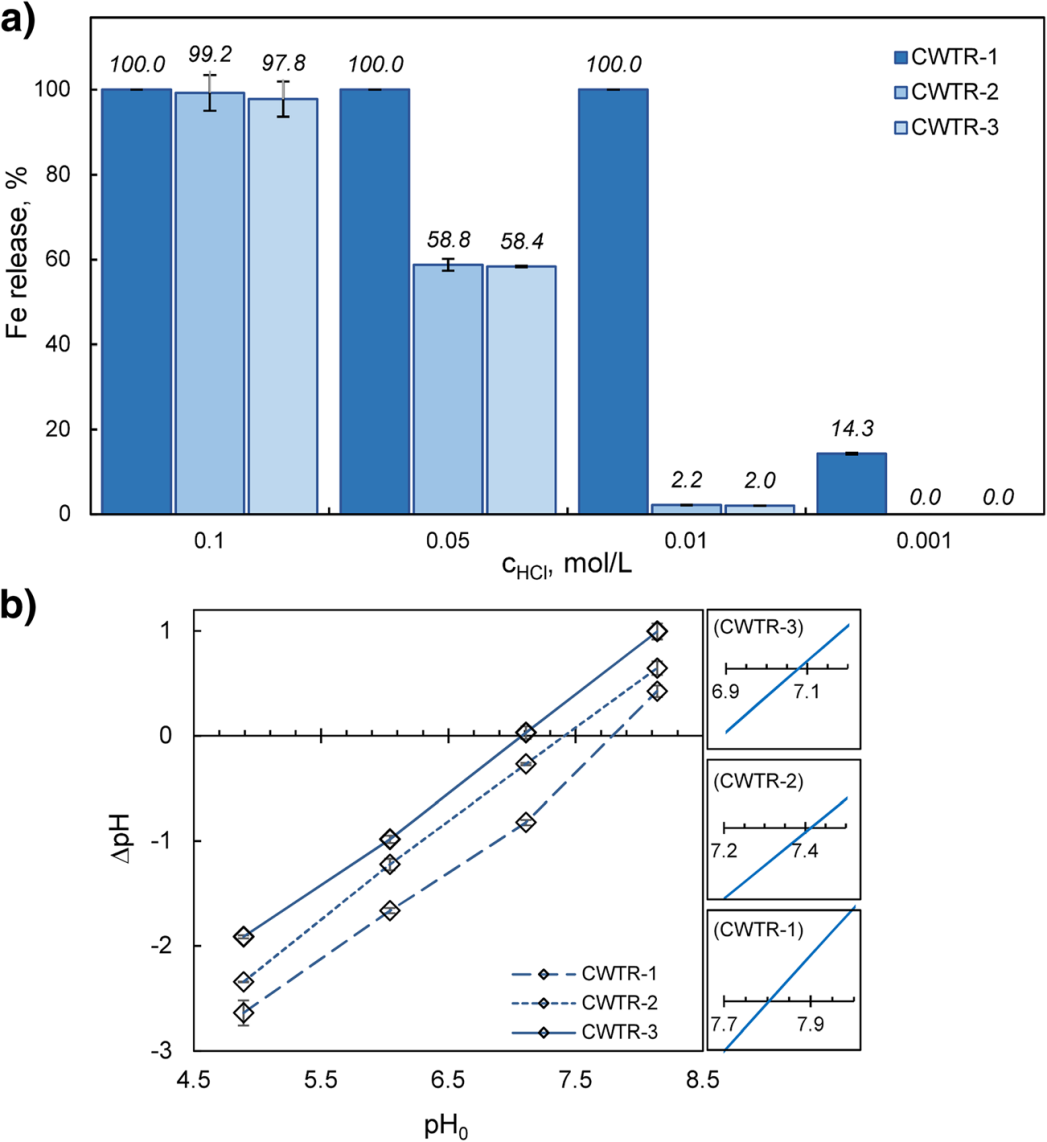
Behavior of selected sorbents in aqueous environment: **a**) durability in HCl solutions, **b**) isoelectric point (pH_pzc_) determination by the drift method

To determine the most suitable product as an arsenic sorbent, the durability under various acidic conditions and the values of the point of zero charge (pH_pzc_) of selected hybrid polymers were determined. As can be seen from the presented results (Fig. 3a), the non-cross-linked product exhibited remarkably low resistance for iron leaching even in 0.001 M HCl, and under more acidic conditions, it underwent a complete dissolution. On the other hand, both cross-linked products showed similar, significantly greater durability under such conditions. Under pH 3, there was no leaching of iron from sorbent beads, and only below pH 2 did the iron loss exceed 2%, increasing to 98–99% in 0.1 M HCl. Nevertheless, even in such an acidic environment, the beads remained undissolved.

The isoelectric point (pH_pzc_) of the individual sorbent is an important property determining its behavior in aqueous solutions under various pH conditions. Below pH_pzc_, the sorbent’s surface exhibits a positive electrical charge, which facilitates adsorption of anions, whereas above pH_pzc_, the effectiveness of anion binding gradually drops. As revealed by Fig. 3b, the cross-linking of the chitosan slightly influenced the point of zero charge of the obtained sorbents. The lower value of pH_pzc_, close to neutral, was determined for the hybrid polymer with the highest cross-linking degree (CWTR-3).

Taking into consideration the shape of the formed beads, their durability in acidic conditions and the lower value of pH_pzc_, the hybrid polymer with the highest cross-linking degree (CWTR-3) was then selected as the most suitable as an arsenic sorbent and subjected to detailed characterization in terms of its composition, structure, and morphology.

**Fig. 4:**
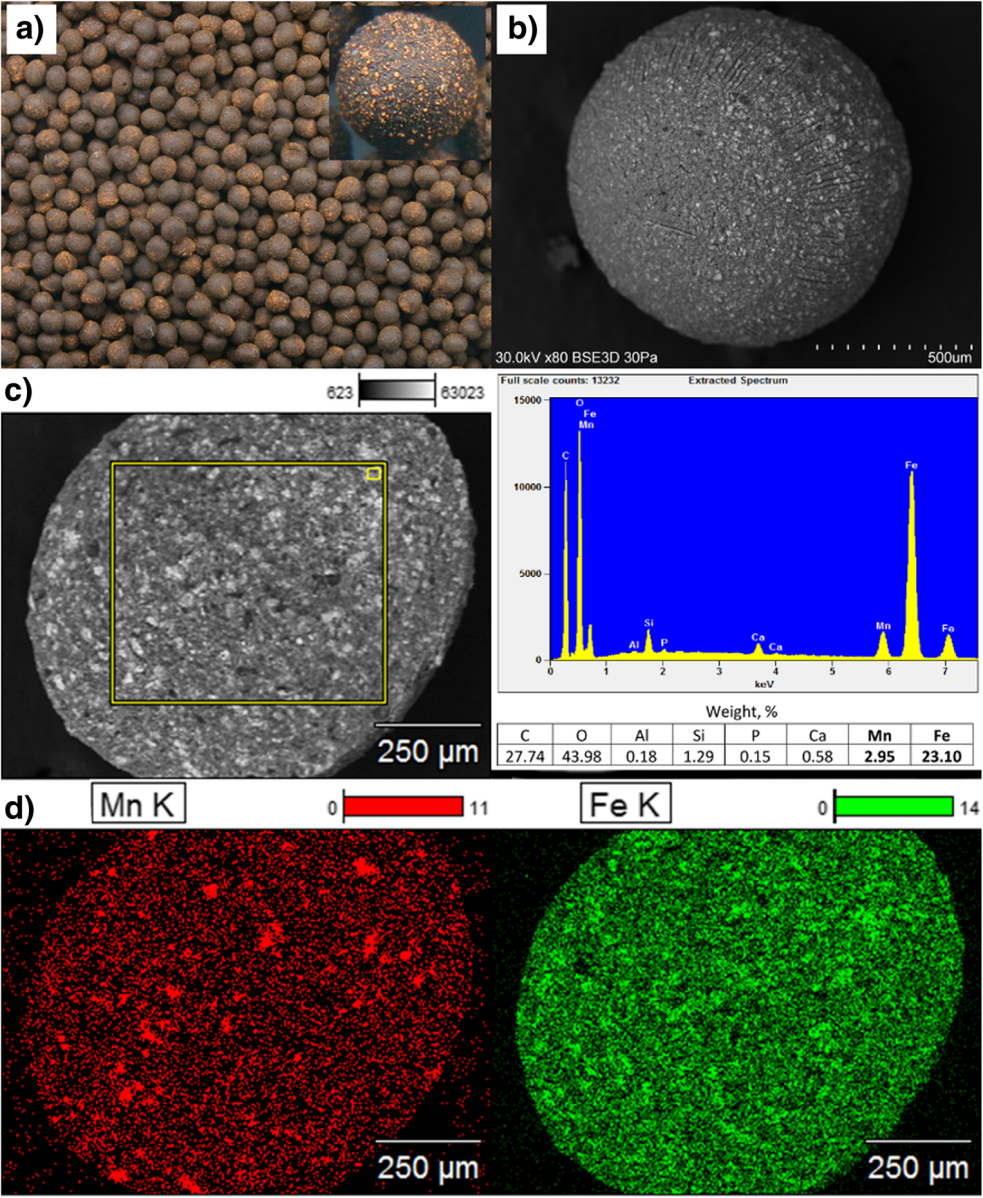
Characterization of the CWTR-3 sorbent: **a**) macro-photography of the beads, **b**) SEM image of the whole bead, **c**) SEM image of the bead’s cross-section with EDS analysis, **d**) SEM EDS mapping of iron and manganese dispersion in the cross-section of a bead

As shown in Fig. 4a–b, the beads of the selected sorbent are characterized by a regular, spherical shape, and rough surface. Simultaneously, both on the bead’s surface and in its cross section, the particles of the inorganic deposit dispersed within the polymer matrix are apparent. EDS analysis results (Fig. 4d) confirmed that iron and manganese oxides are evenly distributed within the sorbent beads. However, the more detailed analysis showed distinctly greater fluctuations of manganese content within the polymer bead compared with iron (Figs. S3 and S4 and Tables S2 and S3). This may result from a different course of iron and manganese precipitation during the water deironing process. Fe(II) is readily oxidized to Fe(III) followed by fast precipitation of iron hydroxide in the bulk of the solution, whereas MnO_2_ is mainly precipitated on the surface of sand particles in the filtration process. Thus, manganese oxide is present in the sludge from filter backwashing in the form of larger particles dispersed within the iron oxides.

**Fig. 5:**
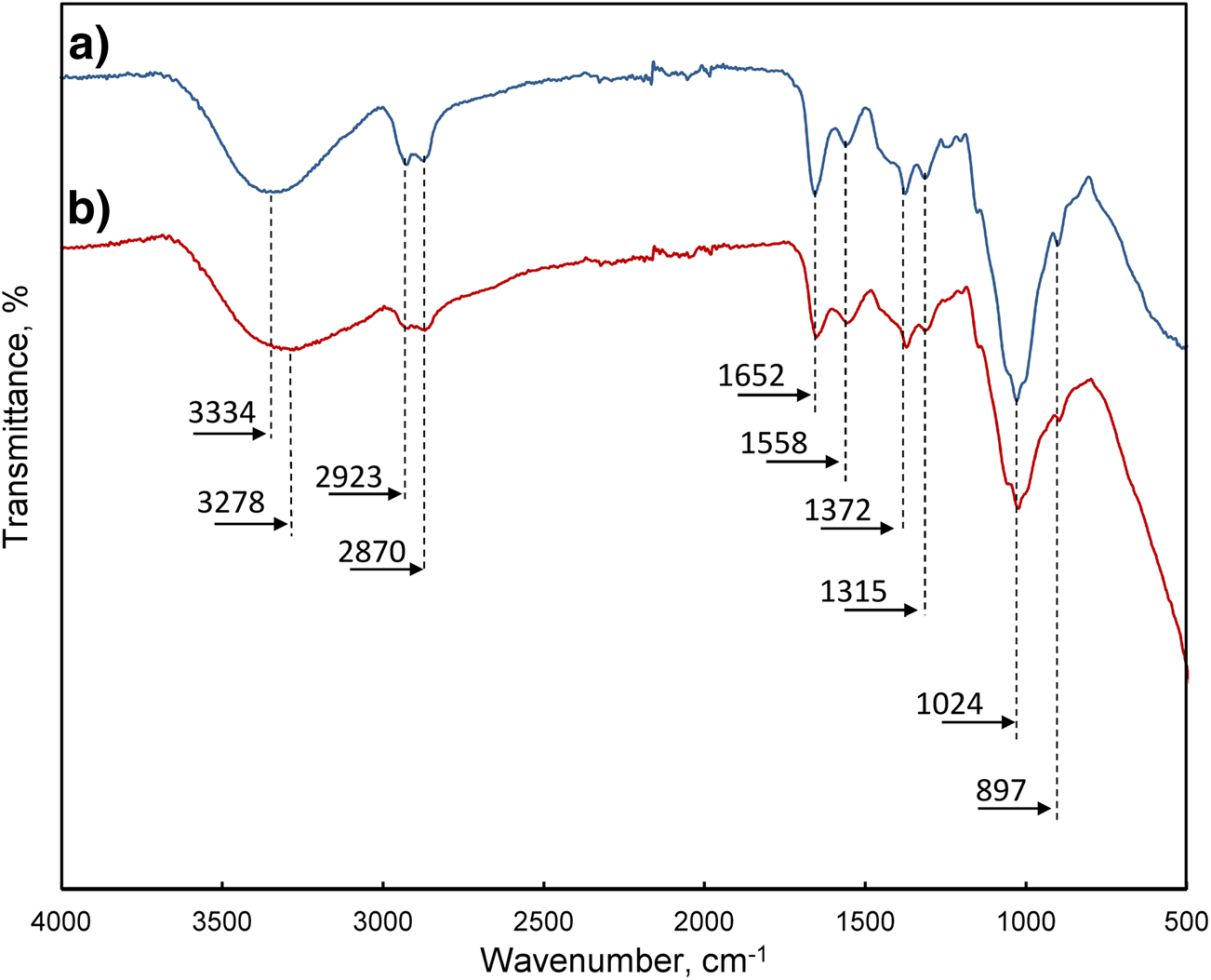
Fourier-transform infrared spectroscopy (FTIR) analysis of the bare cross-linked chitosan and CWTR-3

The Fourier-transform infrared spectroscopy (FTIR) analysis of the bare, cross-linking chitosan and the hybrid polymer confirmed the presence of the characteristic peaks of the chitosan chains and its functional groups in the sorbent (Fig. 5).

The intense peak at 1653 cm^−1^ can be attributed to the amide I stretching vibration of the –NH–CO– band (along with an N–H deformation mode) in acetylated amino groups (Travlou et al. 2013) and to the stretching vibrations of the C=N bond in the imine groups (Gupta et al. 2009; Jóźwiak et al. 2017). The latter is formed as a result of cross-linking reaction between the carbonyl group of glutaraldehyde and the amine group of chitosan. Simultaneous lack of the characteristic band of main nitrogenous functions (–NH_2_) in chitosan at ~ 1600 cm^−1^ confirms the complete cross-linking of the supporting chitosan in CWTR-3 sorbent (Zhang et al. 2018). The bands visible at 1558 and 1312 cm^−1^ could be due to the amide II band and C–N stretching of amide III of chitosan, respectively (Dassanayake et al. 2015). The broadband in the region of 3000–3500 cm^−1^ may be attributed to O–H overlapped to N–H stretching vibrations of the chitosan. Two peaks observed at 2923 and 2870 cm^−1^ may be assigned to stretching vibrations of the C–H bond in methylene and methyl groups, respectively (Silva et al. 2012). The band at 1372 cm^−1^ may be attributed to bending vibrations of C–H in –CH_3_ groups. Peaks at 1025 and 898 cm^−1^ are characteristic for the stretching vibration of C−O−C in the glucose circle and for β-linkage of the polysaccharide, respectively (Travlou et al. 2013; Dassanayake et al. 2015). Entrapment of the inorganic deposit into the chitosan matrix resulted in a decrease of the intensity of peaks attributed to chitosan functional groups and broadening of the peak centered at ~ 3300 cm^−1^ (due to hydroxyl groups of iron oxides and surface water molecules). Moreover, a slight change in the shape of the peak around 988 cm^−1^ is observed, which may be due to bending vibrations of Fe–OH modes of feroxyhyte (Mei et al. 2015).

**Fig. 6:**
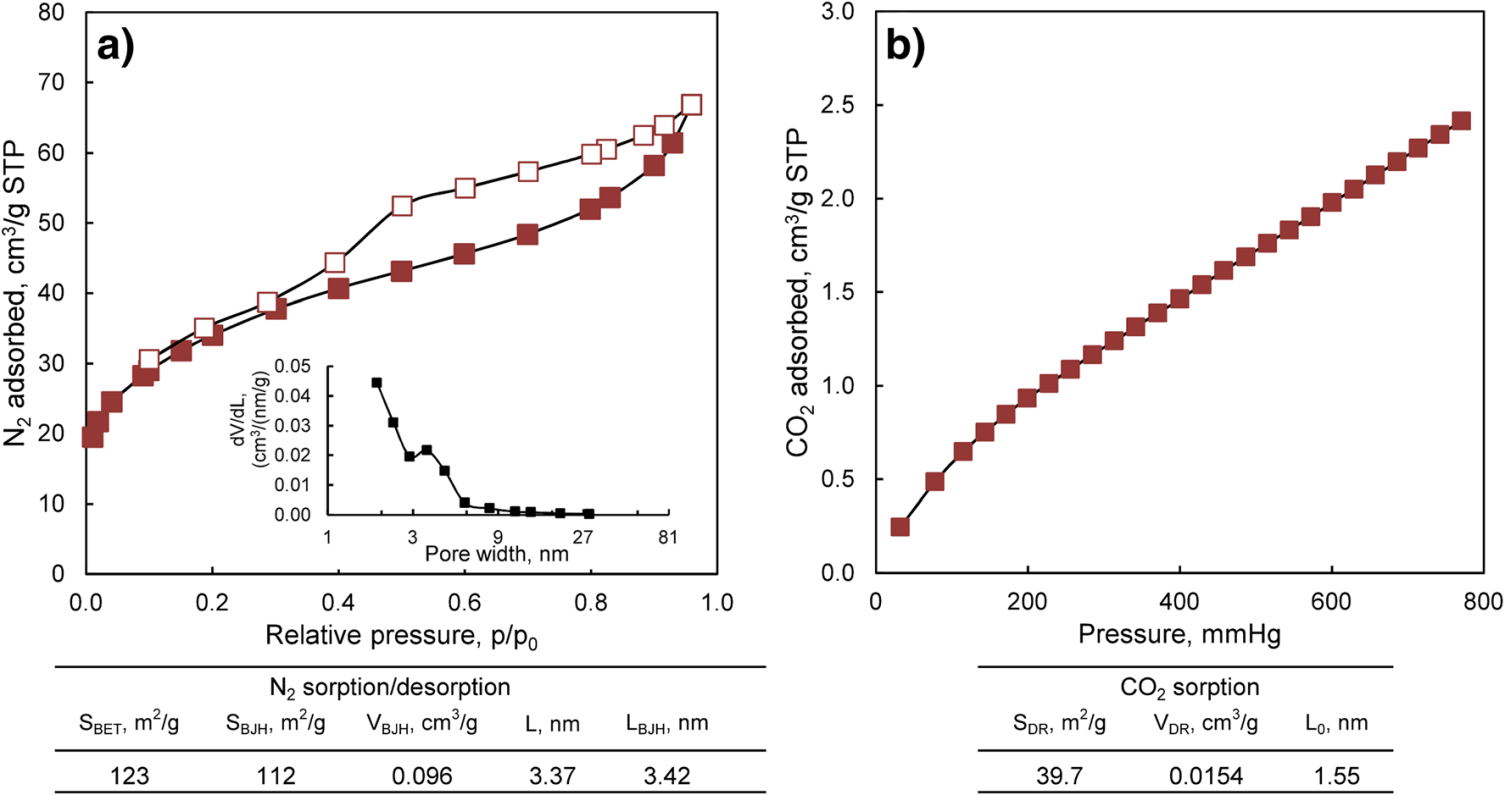
Porous characteristics of the CWTR-3 determined by: **a**) N_2_ sorption/desorption at 77 K, **b**) CO_2_ sorption at 273 K

As it was demonstrated, air-drying of the formed sorbent made it possible to obtain a durable sorbent in the physical form which is suitable for column processes, most useful in waters and wastewater treatment. However, this method led to the destruction of the hydrogel structure of the chitosan, which negatively influenced its spatial structure. The loss of water caused the shrinkage of the sorbent’s beads, resulting in the reduction of their diameter from around 3 to 1 mm. A detailed study of the porous structure of the obtained hybrid polymer was thus conducted and the results are presented in Fig. 6.

The total specific surface area of the sorbent was 123 m^2^/g, and it resulted mainly from the presence of meso- and micropores in the material. The dominant pore diameter determined from the N_2_ adsorption/desorption isotherm using the BJH method was 3.42 nm. The analysis was then complemented using CO_2_ adsorption, a more suitable method for micropore determination, which confirmed the essential contribution of micropores in the sorbent structure, with the dominant pore diameter equal to 1.55 nm (Fig. 6b). The porosity of the hybrid polymer arises from the inorganic deposit content, since the specific surface area of the bare cross-linked chitosan determined from the N_2_ adsorption/desorption isotherm was below 0.1 m^2^/g.

### Kinetics of As(III) and As(V) sorption on CWTR-3

As it is well known from the literature, the total volume of the sorbent’s micropores impacts its total adsorption capacity, whereas the presence of macropores, acting as the transport channels, improves the process kinetics (Birdi 2003; Worch 2012). Thus, sorbents with porosity similar to that described above for CWTR-3 hybrid polymer usually exhibit a high adsorption capacity but also large diffusional constraints, which significantly lengthen the time necessary to achieve an equilibrium. On the other hand, the most important feature of the sorbents used in fixed-bed systems is their ability to reduce the concentration of the removal pollutant below the desired limit. For this purpose, and due to the specificity of the column process, the ratio of the sorbent to the removed species is usually multiplied higher than that in batch mode. As a result, the adsorption sites on the outermost part of the sorbent’s beads are mainly used for binding pollutants. Moreover, since the process is conducted only to the breakthrough point of the sorbent’s bed, the achieved adsorption capacity is usually many times lower than that determined in equilibrium studies.

**Fig. 7:**
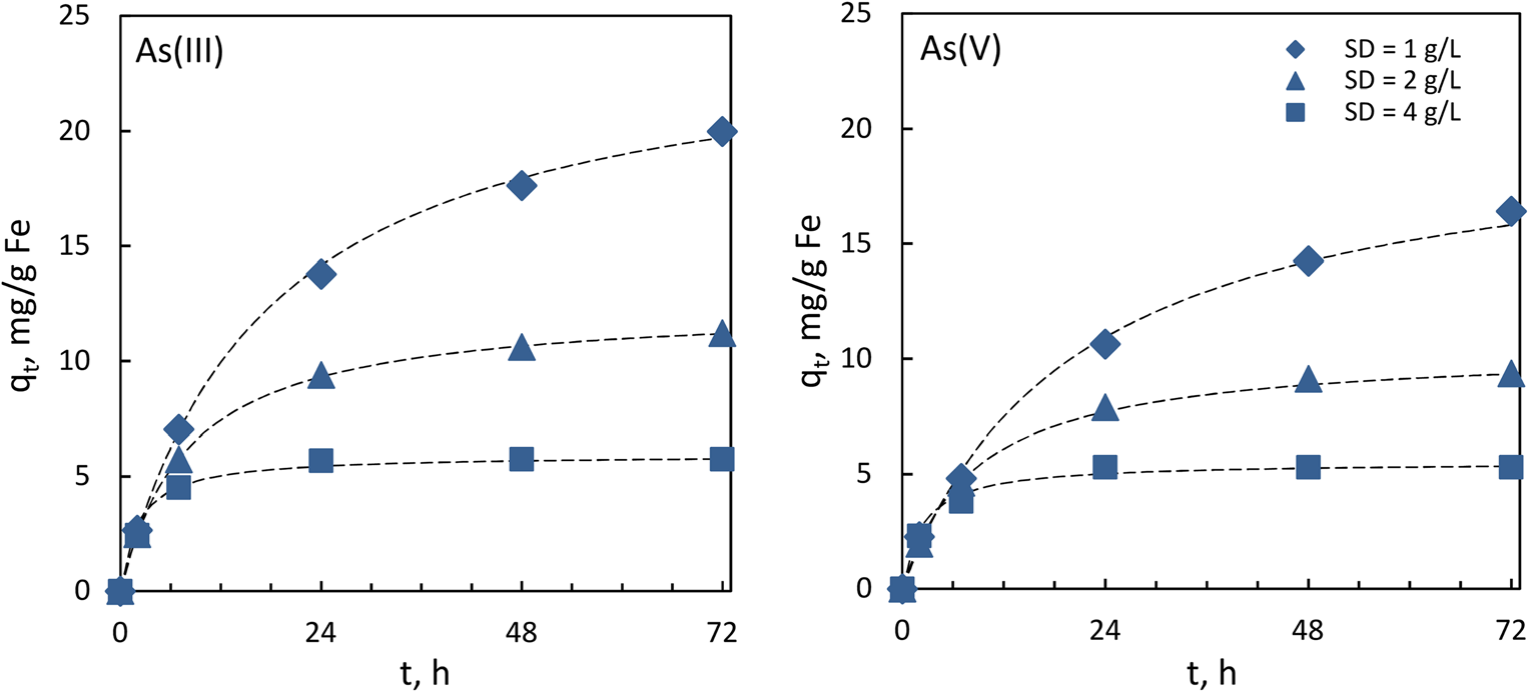
Kinetic curves for As(III) and As(V) adsorption on CWTR-3 at various sorbent dosages: *c*_0_ = 4 mg/L, *t* = 72 h, the dashed lines are plots of pseudo-second-order kinetic model

To examine the kinetic properties of the formed sorbent and its capacity for deep removal of arsenates and arsenites, kinetic studies were conducted using various sorbent dosages (1, 2, and 4 mg/L). The results are presented in Fig. 7 and Table 3.

**Table 3:** PSO kinetic parameters for arsenic adsorption on dried CWTR-3 at various sorbent dosages

Sorbent dosage, g/L	As(III)					As(V)				
	*R* ^2^	*q*_m_, mg/g	*q*_exp_, mg/g	*k*_2_, g/(mg·h)	*h*_0_, mg/(g·h)	*R* ^2^	*q*_m_, mg/g	*q*_exp_, mg/g	*k*_2_, g/(mg·h)	*h*_0_, mg/(g·h)
1.0	0.998	24.54	19.98	0.002	1.40	0.990	20.32	16.42	0.002	0.99
2.0	1.0	12.46	11.21	0.010	1.54	0.999	10.46	9.35	0.011	1.22
4.0	0.999	5.93	5.75	0.078	2.73	0.999	5.52	5.30	0.075	2.30

As can be seen, the highest sorbent dosage allowed reduction of the arsenic concentration (for both As(III) and As(V)) below the detection limit (0.1 mg/L) within a few hours. It is worth noting that such a low concentration of species being removed in the solution after adsorption in batch mode reveals the capacity of the sorbent for deep adsorption. The studied hybrid polymer, aside from the inorganic deposit providing adsorptive properties, contains imine functional groups which undergo protonation, thereby facilitating the diffusion of negatively charged arsenates to active sites on the sorbent surface. For arsenites, the adsorption process is additionally enhanced by earlier discussed reductive dissolution of MnO_2_ (Eq. 1). However, decreasing the sorbent dosage resulted in significant elongation of time needed to achieve equilibrium. For the lowest amount of the sorbent, the adsorption process was not finished even after 72 h. It is well known that for sorbents in the form of granules, in contrast to microspheres, the main rate-limiting step of adsorption is intraparticle diffusion and that the process rate is inversely proportional to the square of the particle radius (Badruzzaman et al. 2004). This is also confirmed by the shapes of the presented kinetic curves and highly diverse kinetic constants (Table 3). The best results obtained for the highest sorbent dosage might arise from binding of arsenic mainly on the readily accessible sites on the outer surface of the beads, which reduced the intraparticle diffusion. At lower sorbent dosage, however, these active sites became completely occupied and the remaining arsenate molecules diffused into the beads, which markedly diminished the overall adsorption rate. On the other hand, better utilization of the active sites at lower sorbent dosages resulted in higher total adsorption capacity. It is noteworthy that the internal structure of the sorbents based on hydrogel formation (e.g., chitosan, alginate) might be substantially improved by replacing air-drying with freeze-drying or drying in supercritical carbon dioxide (scCO_2_) (Błaszczyński et al. 2013; Wang et al. 2014). As a result, products characterized by a significantly more porous structure (aerogels) are formed. Nonetheless, besides a significant increase in the costs of the process, the intraparticle diffusion still remarkably influences the rate of adsorption (Ociński et al. 2019). Meanwhile, the main goal of this study was to optimize the formation process of the low-cost sorbent on the basis of an industrial by-product, with the capacity for deep adsorption of arsenic. Notwithstanding, it would be of great interest to investigate whether the replacement of air-drying with freeze-drying or drying in scCO_2_ would be economically viable in view of prospective applications of this sorbent.

## Conclusions

Utilization of the waste sludge from water deironing by its dispersion within the cross-linking chitosan supporting polymer allowed one to obtain an effective arsenic sorbent in the form of spherical beads. The proper combination of the individual parameters of the ex situ formation process was crucial to achieve the hybrid polymer with the adsorptive properties and the physical form and durability desired in column adsorption systems. The optimal values of the parameters were as follows: chitosan polymer concentration 1.5% *w*/*w*, Mn-WTRs to chitosan ratio 1.67, and glutaraldehyde to chitosan amine groups molar ratio 3:1. Cross-linking of the polymeric support had almost no effect on the adsorptive properties of the product, but remarkably improved its durability in an acidic environment. Despite the micro- and mesoporous structure and the lack of macropores resulting in high intraparticle diffusion resistance, deep adsorption of arsenite and arsenate was possible even in batch mode by using the proper sorbent dosage. The results of preliminary adsorption experiments showed the prospective usefulness of the formed sorbent for deep removal of arsenic in fixed-bed adsorption systems. However, further research is essential to confirm the ability of the formed sorbent to mitigate the arsenic concentration in aqueous solutions below 0.01 mg/L (maximum contaminant level for arsenic in drinking water) in column mode, and the possibility of its regeneration and reuse.

## Electronic supplementary material

ESM 1 (PDF 1035 kb)
