# Supplementary material for: Optimization of hybrid polymer preparation by ex situ embedding of waste Fe/Mn oxides into chitosan matrix as an effective As(III) and As(V) sorbent
Source: Environ Sci Pollut Res Int. 2019 Jul 5;26(25):26026–38. doi: 10.1007/s11356-019-05856-x (PMC6717189; doi:10.1007/s11356-019-05856-x)
Supplement: Supplementary file 1 — (PDF 1035 kb) [file 11356_2019_5856_MOESM1_ESM.pdf]

Supplementary Material

Optimization of hybrid polymer preparation by *ex-situ* embedding of waste Fe/Mn oxides into chitosan matrix as an effective As(III) and As(V) sorbent

Daniel Ociński

Department of Industrial Chemistry, Wroclaw University of Economics, ul. Komandorska 118/120, 53-345 Wrocław, Poland (daniel.ocinski@ue.wroc.pl, tel.: +48 71 3680276)

Table S1. Equations of kinetic adsorption models

Model	Equation	Parameters
Pseudo-first order	$dq_t /dt = k_1(q_e - q_t)$<br><br>$\ln(q_e - q_t) = \ln q_e - kt$<br>*(integrated form)*	k_1, k_2 – equilibrium rate constants of the pseudo-first and pseudo-second kinetic adsorption models,<br><br>q_e – amount of adsorbate adsorbed at equilibrium,<br><br>q_t – amount adsorbed at time t,
Pseudo-second order	$dq_t/dt = k_2(q_e - q_t)^2$<br><br>$t/q_t = 1/k_2q_e^2 + t/q_e$<br>*(integrated form)*<br><br>$h_0 = k_2q_e^2$ – initial sorption rate	

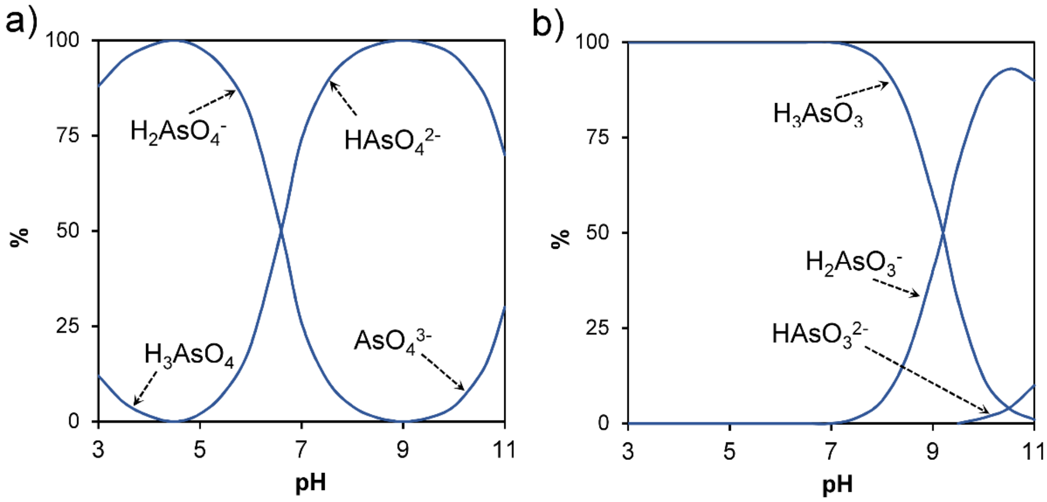

Fig. S1. Arsenate (a) and arsenite (b) speciation as a function of pH

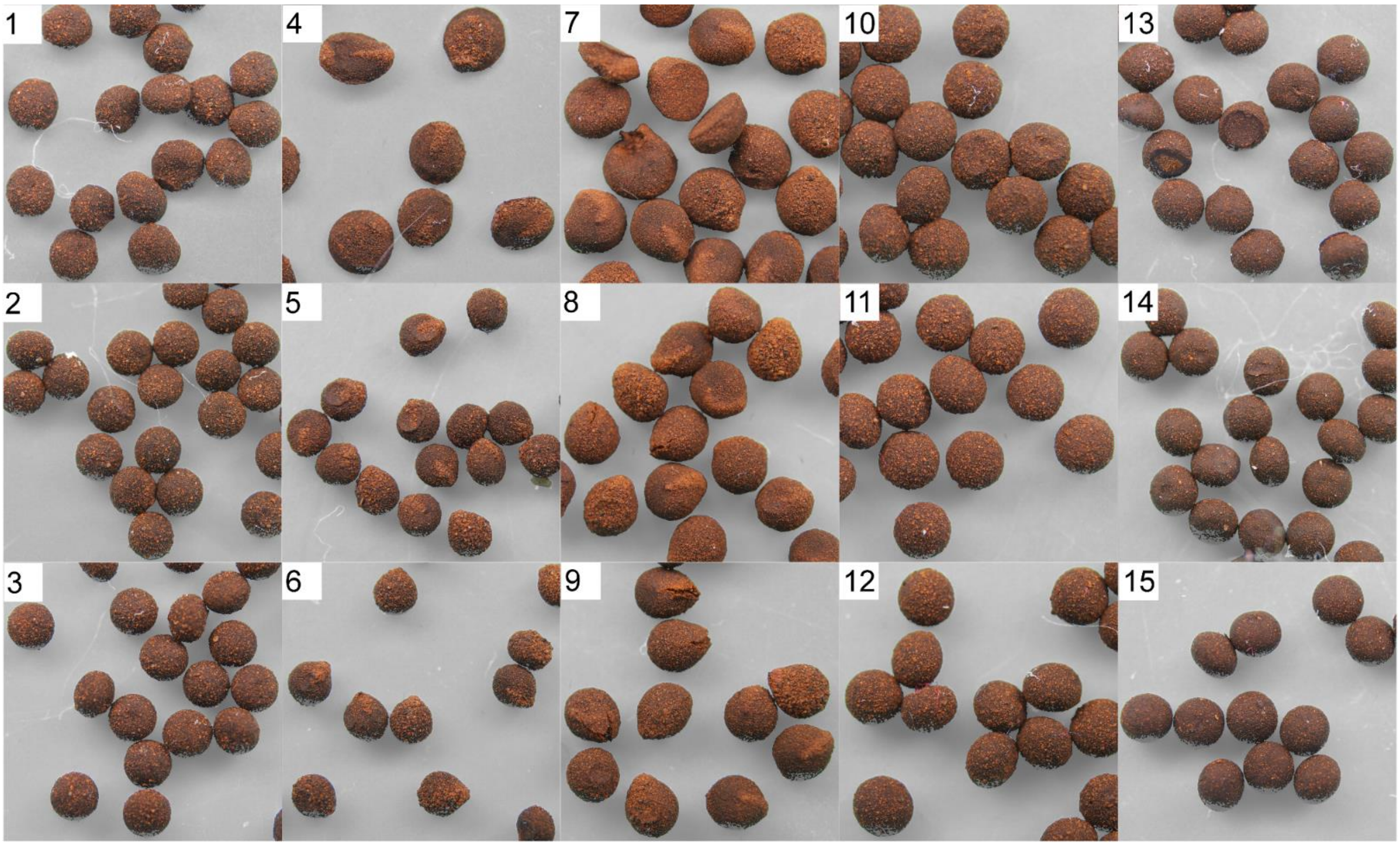

Figure S2. Macro photographs of the air-dried products CWTR-1 to CWTR-15

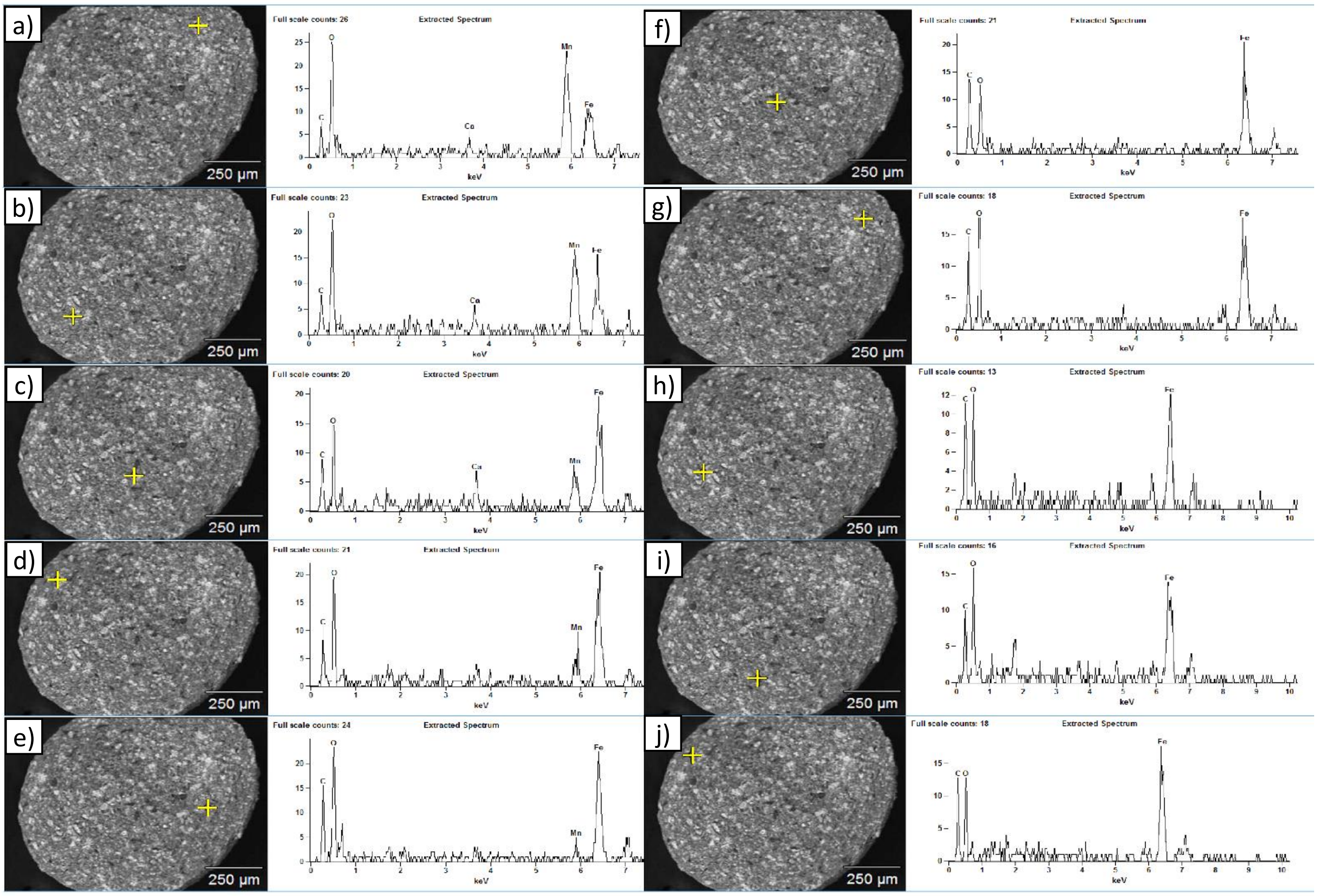

Figure S3. Spot EDS analysis results of the bead's cross-section

Table S2. Fe and Mn content in CWTR-3 according to point EDS analysis

Point	Fe content, %	Mn content, %
a	14.85	26.08
b	12.78	22.07
c	25.46	10.25
d	24.56	8.9
e	24.42	2.64
f	24.01	-
g	28.61	-
h	22.26	-
i	30.9	-
j	31.08	-

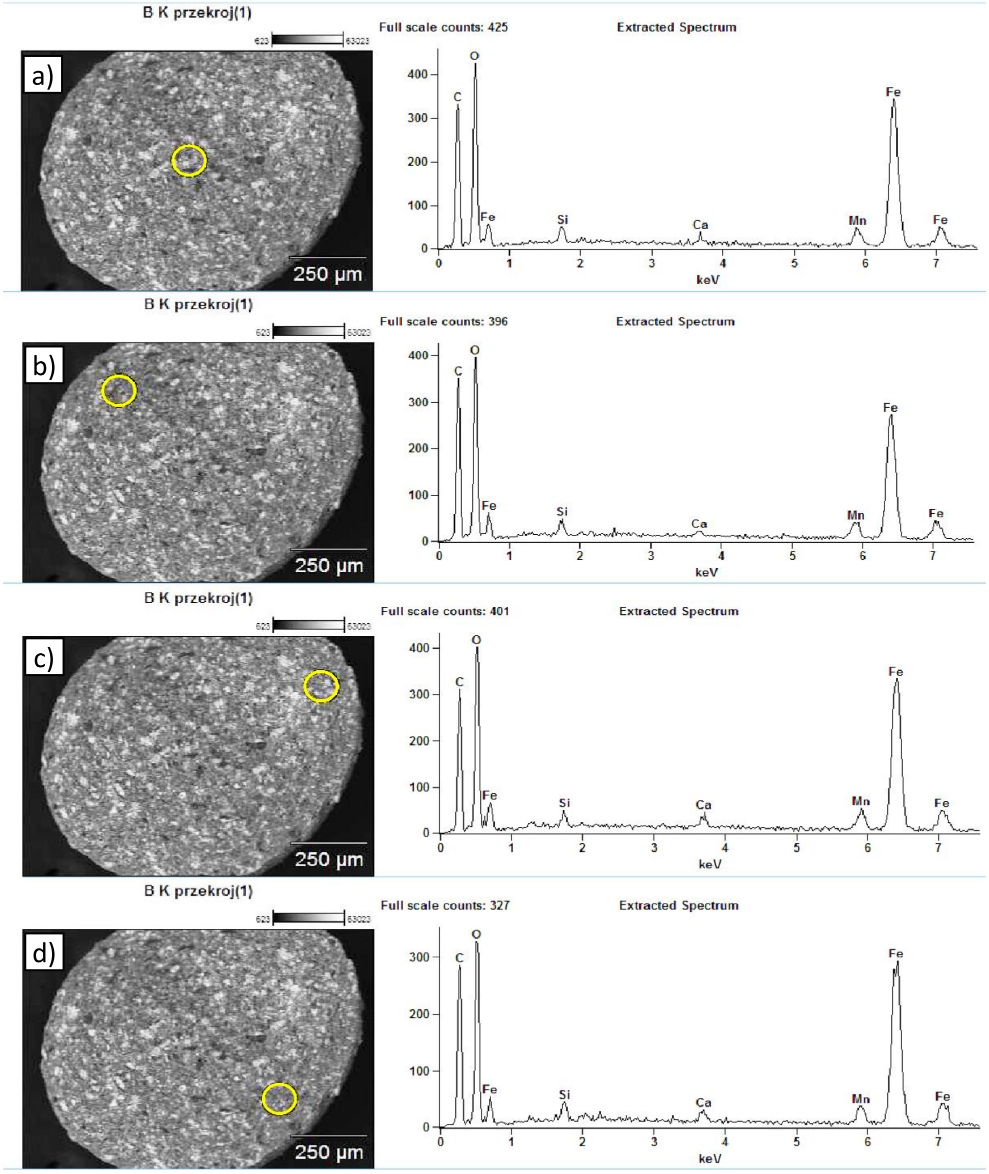

Figure S4. Area EDS analysis results of the bead's cross-section.

Table S3. Fe and Mn content in CWTR-3 according to area EDS analysis

Area	Fe content, %	Mn content, %
a	22.26	2.24
b	19.38	2.33
c	24.68	2.99
d	23.96	2.34
